# The Asiatic Cholera in Persia

**Published:** 1847-02

**Authors:** 


*The Asiatic Cholera in Persia.*—According to the Gazette Médicale, six Princes and several Princesses of the Court of Persia have been cut off by the Asiatic cholera. The mother of the Prince Royal, and the only daughter of the Schah, had been attacked, but had recovered under the treatment of Dr. Cloquet. Among the victims is the celebrated Mirza-Aboul-Assan-Khan, minister of foreign affairs, —who was ambassador to this country in the year 1820. Another minister of the Schah, the Visier of the Prince Royal, and other high functionaries of the Court, have also been cut off by cholera. The disease appears to have been particularly fatal among the upper classes. It was spreading in all directions, and had taken the course of Astrachan and Moscow. It was expected, however, that its progress would be arrested by the cold of winter.—*London Med. Gaz.*

